# Gynecomastia and Its Management In Boys With Partial Androgen Insensitivity Syndrome

**DOI:** 10.1210/clinem/dgae562

**Published:** 2024-08-30

**Authors:** Supitcha Patjamontri, Angela K Lucas-Herald, Jillian Bryce, Erica van den Akker, Martine Cools, Evgenia Globa, Gil Guerra-Junior, Olaf Hiort, Paul Hofman, Paul-Martin Holterhus, Ieuan A Hughes, Anders Juul, Anna Nordenstrom, Gianni Russo, Marianna R Stancampiano, Sumudu N Seneviratne, Rieko Tadokoro-Cuccaro, Ajay Thankamony, Naomi Weintrob, Natalia Zelinska, S Faisal Ahmed

**Affiliations:** Developmental Endocrinology Research Group, University of Glasgow, Royal Hospital for Children, Glasgow G51 4TF, UK; Division of Endocrinology and Metabolism, Department of Pediatrics, Faculty of Medicine Siriraj Hospital, Mahidol University, Bangkok 10700, Thailand; Developmental Endocrinology Research Group, University of Glasgow, Royal Hospital for Children, Glasgow G51 4TF, UK; Developmental Endocrinology Research Group, University of Glasgow, Royal Hospital for Children, Glasgow G51 4TF, UK; Division of Pediatric Endocrinology, Department of Pediatrics, Sophia Children's Hospital and Center of Expertise DSD, Erasmus University Medical Center, Rotterdam 3000 CA, Netherlands; Department of Paediatric Endocrinology, Ghent University Hospital, University of Ghent, Ghent 9000, Belgium; Ukrainian Scientific and Practical Center of Endocrine Surgery, Transplantation of Endocrine Organs and Tissues of the Ministry of Health of Ukraine, Kyiv 01021, Ukraine; Interdisciplinary Group for the Study of Sex Determination and Differentiation, School of Medical Sciences (FCM), State University of Campinas, Campinas 13083-887, Brazil; Division of Paediatric Endocrinology and Diabetes, Department of Paediatrics and Adolescent Medicine, University of Lübeck, 23538 Lübeck, Germany; Liggins Institute, University of Auckland, 1023 Auckland, New Zealand; Division of Pediatric Endocrinology and Diabetes, Department of Pediatrics, University Hospital of Schleswig-Holstein, Campus Kiel/Christian-Albrechts University of Kiel, 24105 Kiel, Germany; Department of Paediatrics, University of Cambridge, Cambridge CB2 0QQ, UK; Growth and Reproduction Rigshospitalet, University of Copenhagen, DK-2100 Copenhagen, Denmark; Department of Women's and Children's Health, Karolinska Institutet, 171 77 Stockholm, Sweden; Department of Pediatrics, Endocrine Unit, Scientific Institute San Raffaele, Milan 20132, Italy; Department of Pediatrics, Endocrine Unit, Scientific Institute San Raffaele, Milan 20132, Italy; Faculty of Medicine, University of Colombo, Colombo 8, Sri Lanka; Department of Paediatrics, University of Cambridge, Cambridge CB2 0QQ, UK; Department of Paediatrics, University of Cambridge, Cambridge CB2 0QQ, UK; Endocrinology and Diabetes Unit, Dana-Dwek Children's Hospital, Tel Aviv 6423906, Israel; Ukrainian Scientific and Practical Center of Endocrine Surgery, Transplantation of Endocrine Organs and Tissues of the Ministry of Health of Ukraine, Kyiv 01021, Ukraine; Developmental Endocrinology Research Group, University of Glasgow, Royal Hospital for Children, Glasgow G51 4TF, UK

**Keywords:** androgen insensitivity syndrome, gynecomastia, disorders of sex development, I-DSD registry

## Abstract

**Introduction:**

Partial androgen insensitivity syndrome (PAIS) is a rare condition that is reported to be commonly associated with gynecomastia in males.

**Objectives:**

To assess the management of gynecomastia in male PAIS.

**Materials and Methods:**

Retrospective review of males with PAIS over the age of 10 years in the I-DSD registry.

**Results:**

Of the 205 eligible cases, information was available for 57 from 13 centers. An androgen receptor gene variant was confirmed in 45 (79%) with a median age at first presentation of 1.0 year (range 0.1, 26.0). Of the 45 genetically confirmed cases, gynecomastia was present in 41 (91%) with a median age at the time of gynecomastia development of 13.5 years (11.0, 29.0). In the other 4 (9%) with no gynecomastia, the median age at last assessment was 15.7 years (10.6, 17.0). In 30 cases with information available, micropenis was present at the time of gynecomastia development in 23 (77%). Of the 35 with information available, 2 (6%) exhibited spontaneous resolution between the ages of 15 and 21 years and 25 (71%) had breast surgery at a median age of 15.7 years (14.0, 23.0). Of these 25, 9 (26%) had previously received medical therapy. The median clinician score of effectiveness for medical therapy was 3 (1, 8) compared to 10 (3, 10) for surgery (*P* < .0001). In 31 with information available, 13 (42%) had received psychology support.

**Conclusion:**

Gynecomastia is common in PAIS but not universal. Surgical management may be more effective than medical therapy, but there is a need for further standardized and systematic studies.

Gynecomastia refers to an enlargement of the male breast caused by benign proliferation of the gland ducts and stromal components including fat (1) and may develop due to an imbalance of estrogen and androgen activity in breast tissue. Physiological gynecomastia is often encountered in early puberty in boys with a prevalence ranging between 20% and 70% (2) and is mostly self-limiting, resolving later in puberty. The average duration of pubertal gynecomastia is about 2 years and coincides with the pubertal rise in systemic testosterone concentrations (3). Postpubertally, 3% of young men may continue to exhibit gynecomastia (4). However, persistent and severe pubertal gynecomastia is more likely to be associated with pathological disorders such as those that affect gonadal function, androgen synthesis, or androgen action (5). Gynecomastia is a prominent clinical feature in boys and men with partial androgen insensitivity syndrome (PAIS) due to a pathological genetic variant in the androgen receptor gene (*AR)* and may be the presenting feature in adolescents with PAIS (6, 7). Studies also suggest that it may be universal in those adolescents with PAIS who present with atypical genitalia in infancy (8). Persistent gynecomastia can have a substantial psychosocial impact on the adolescent (9), and therapeutic options include hormonal manipulation or surgical therapy (2). As PAIS is very rare (10, 11), there is scarce systematic information on the natural history of gynecomastia or its management. Previously, the I-DSD Registry has shown that it is a valuable resource for cases of androgen insensitivity syndrome (10, 12, 13), and the objective of the current study was to explore the development and progression of gynecomastia as well as its management in PAIS through the I-DSD registry platform.

## Patients and Methods

All 46 XY boys and men registered as having PAIS who were over the age of 10 years at the time of data collection were identified in the I-DSD Registry (https://sdmregistries.org/), an international database of information on individuals with differences or disorders of sex development and where data are deposited by their physicians after obtaining consent from the patients or their guardians. The registry is approved by the National Research Ethics Service (19/WS/0131) in the United Kingdom as a research database of information that is collected as part of routine clinical care. All centers with eligible cases in the I-DSD Registry were approached in 2020, and those centers that agreed to participate contributed data to the study between September 2020 and October 2021. Data that were collected included clinical characteristics at first presentation, details on *AR* genetic testing, age at onset of puberty, age at onset of gynecomastia development, biochemical profiles at onset of gynecomastia, and details of the management of gynecomastia. PAIS was only considered in those cases that had a genetically confirmed variant in *AR.* The external masculinization score (EMS) was calculated as previously described (14). Briefly, the EMS is a composite score that is based on the reported site of the urethral meatus, the location of the gonads, the presence of a micropenis, and the presence of labioscrotal fusion; normal male external genitalia as would be expected in a boy would have a score of 12, whereas normal female external genitalia would have a score of 0. Because of differences in assays, time of measurement, and center-specific reference values, hormone levels were interpreted by the reporting center using the center's in-house reference values as low/normal/high for age, with normal, high, and low defined as hormone concentrations within normal, above the 95th percentile, and below the 5th percentile of reference rages for age and sex, respectively. Lastly, the reporting clinician was asked to rate the effectiveness of any therapy administered for the management of gynecomastia on a 10-point numerical rating scale that was been created for the purpose of this study and where 10 represented maximum effectiveness. The effectiveness scores for surgery and drug therapy were defined as 1, no reduction in gynecomastia; 2, minimal reduction in gynecomastia; 3, slight reduction in gynecomastia; 4, moderate reduction in gynecomastia; 5, noticeable reduction in gynecomastia; 6, considerable reduction in gynecomastia; 7, significant reduction in gynecomastia; 8, strong reduction in gynecomastia; 9, very strong reduction in gynecomastia; 10, maximum reduction in gynecomastia. The effectiveness scores for clinical psychology were defined as 1, no benefit of intervention; 2, minimal benefit of intervention; 3, slight benefit of intervention; 4, moderate benefit of intervention; 5, noticeable benefit of intervention; 6, considerable benefit of intervention; 7, significant benefit of intervention; 8, strong benefit of intervention; 9, very strong benefit of intervention; 10, maximum benefit of intervention. Continuous variables were described as medians and ranges, and intergroup comparison for these variables was performed by the Mann–Whitney U test. The association between categorical variables was assessed by Fisher's exact test. All statistical analyses were performed using SPSS Statistics version 20 (IBM Corporation, Armonk, New York, USA), and a *P*-value of less than .05 was considered statistically significant.

## Results

### Molecular Genetic Confirmation

**Figure 1.**
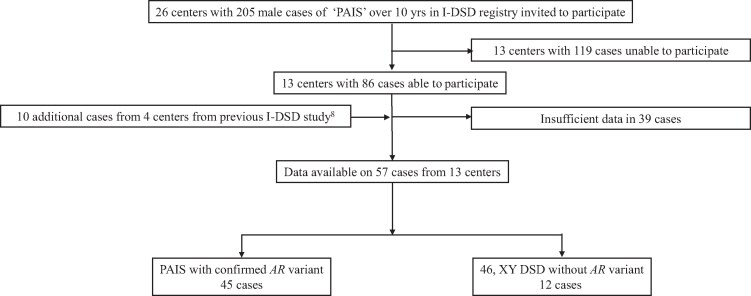
Consort diagram describing the recruitment of partial androgen insensitivity syndrome cases from the I-DSD registry.

Of the 205 cases reported as PAIS in the I-DSD Registry from 26 centers and who were over 10 years of age, clinical information was available for final analysis for 57 cases from 13 centers with a median number of cases per center of 3 (range 1, 14) (Fig. 1). Of these 57 reported cases of PAIS, the diagnosis had been genetically confirmed in 45 (79%) with 32 different *AR* variants (Table 1) of which 4 (12%) were located in the N-terminal domain (NTD), 7 (22%) in the DNA-binding domain (DBD), and the remaining 21 (66%) in the ligand-binding domain (LBD).

**Table 1.** Summary of clinical characteristics, *AR* variants, and the management of Gy’mastia in 46 partial androgen insensitivity syndrome cases

	*AR* variant	Age at initial present^n^	Clinical features	EMS at presentation	Age at Gy’mastia	Gy’mastia therapy	Drug	Age At Starting Drug (years)	Drug Duration (months)	Effectiveness Score for Drug	Age at Surgery (years)	Effectiveness Score for Surgery	Age at Psychology (years)	Effectiveness Score for Psychology
Gy’mastia														
1	D565N	0.1	G, H, M	3.0	14.0	Sx	NA	NA	NA	NA	15.0	10	NA	NA
2*^a^*	D565N	0.1	G, H	NK	NK	Not known	NK	NK	NK	NK	NK	NK	NA	NA
3	F755L	0.1	G, H, M	3.0	15.0	Drug -> Sx	Tamoxifen 10 mg	15.0	0.5	1	17.0	10	NA	NA
4*^a^*	Q868L*	0.1	G, H, M	6.0	12.0	Drug -> Sx, Psy	Letrozole 2.5 mg	12.0	NK	6	17.3	9	12.0	7
5*^a^*	L713F	0.1	B, G, H	9.0	NK	Sx	NA	NA	NA	NA	NK	NK	NA	NA
6*^a^*	L713F	0.1	G, M	9.0	NK	Sx	NA	NA	NA	NA	14.0	NK	NA	NA
7	L839I*	0.1	G, H, M	6.0	13.0	Not known	NK	NK	NK	NK	NK	NK	NA	NA
8	V904M	0.1	G, H, M	6.0	12.7	None, Psy	NA	NA	NA	NA	NA	NA	NK	NK
9*^a^*	c.1769–17_1769–16dup2 kb (exon 04)*	0.1	G, H, M	3.0	13.5	Sx, Psy	NA	NA	NA	NA	16.0	3	NK	NK
10	S598R, E210E*	0.1	G, H, M	3.0	13.8	Drug, Psy	Letrozole 2.5 mg	13.8	12	NK	NA	NA	NK	NK
11	S814I*	0.1	G, M	9.0	12.7	Drug -> Sx, Psy	DHT gel	12.7	6	1	15.3	10	13.2	4
12*^a,b^*	R608Q	0.1	G, H, M	3.0	13.0	None, Psy	NA	NA	NA	NA	NA	NA	14.0	5
13*^a,b^*	R608Q	0.1	G, H, M	3.0	13.4	None, Psy	NA	NA	NA	NA	NA	NA	14.5	4
14*^a^*	I843T	0.3	G, M	NK	12.0	Sx, Psy	NA	NA	NA	NA	14.0	NK	NK	8
15	Q712E	0.5	G, H, M	NK	11.0	Drug -> Sx	Tamoxifen 10 mg	13.0	49	6	awaiting	NA	NA	NA
16	L548F	0.6	G, H, M	8.0	12.8	Drug -> Sx	Anastrozole 1 mg	15.2	15	3	16.4	10	NA	NA
17	F813C*	0.8	G, H, M	5.0	13.2	None, Psy	NA	NA	NA	NA	NA	NA	NK	NK
18	V676A*	0.9	G, M	9.0	28.2	None	NA	NA	NA	NA	NA	NA	NA	NA
19*^a^*	R856H	1.0	B, G, H, M	2.0	NK	Sx	NA	NA	NA	NA	14.0	NK	NA	NA
20*^a^*	R856H	1.0	B, G, H, M	2.0	NK	Sx	NA	NA	NA	NA	15.0	NK	NA	NA
21	Q799E	6.0	G, H, M	NK	11.0	Sx	NA	NA	NA	NA	17.0	10	NA	NA
22*^a^*	Q825K	7.0	G, M	12.0	14.5	Drug -> Sx	TTD 40 mg	15.5	NK	NK	16.0	9	NA	NA
23	Q2Q*	7.6	G	12.0	11.3	Sx, Psy	NA	NA	NA	NA	awaiting	NA	11.4	4
24*^a^*	Q825K	10.0	G, M	9.0	14.0	Drug	TTD 40 mg	14.0	2	5	NA	NA	NA	NA
25	A699T*	11.0	G	12.0	13.0	Sx	NA	NA	NA	NA	awaiting	NA	NA	NA
26*^a^*	R841H	11.0	B, G, H, M	6.0	12.5	Drug -> Sx, Psy	DHT gel	13.0	3	1	15.0	10	NK	NK
27	P695S*	11.5	G, M	9.0	11.5	Sx, Psy	NA	NA	NA	NA	18.0	10	11.0	7
28	A597T	12.8	G, H, M	6.0	12.8	Drug, Psy	Letrozole 2.5 mg	13.3	ongoing	8	NA	NA	12.8	8
29*^a^*	A597T	13.3	G, H, M	6.0	13.0	Not known	NK	NK	NK	NK	NK	NK	NA	NA
30*^a^*	S244P*	14.0	G, H	9.0	29.0	None	NA	NA	NA	NA	NA	NA	NA	NA
31*^a^*	N757S	14.0	G	NK	14.0	Sx	NA	NA	NA	NA	15.0	10	NA	NA
32*^a^*	N757S	14.0	G	12.0	14.0	Sx	NA	NA	NA	NA	15.0	10	NA	NA
33	Q825K	16.0	G, M	9.0	14.0	Drug -> Sx	TTD 40 mg	18.0	NK	NK	19.0	10	NA	NA
34	R607E*	16.3	G, M	NK	17.7	None	NA	NA	NA	NA	NA	NA	NA	NA
35	Q825K	16.3	G	12.0	NK	Not known	NK	NK	NK	NK	NK	NK	NA	NA
36	R841C	19.0	G, H, M, U	5.5	NK	Not known	NK	NK	NK	NK	NK	NK	NA	NA
37	R630W	20.0	G, H, M	3.0	15.0	Sx	NA	NA	NA	NA	15.0	NK	NA	NA
38*^a^*	Q825K	21.0	G	12.0	15.0	Drug -> Sx	Tamoxifen 20 mg	21.0	18	1	21.0	10	NA	NA
39*^a^*	Q825K	21.0	G, M	9.0	15.0	Sx	NA	NA	NA	NA	23.0	NK	NA	NA
40*^a^*	R789S	26.0	G	9.0	15.0	Not known	NK	NK	NK	NK	NK	NK	NA	NA
41	R841H	NK	G, M, U	NK	13.5	Sx	NA	NA	NA	NA	16.0	9	NA	NA
No Gy’mastia														
42*^a^*	S598R	0.1	H, M	NK	NA	None	NA	NA	NA	NA	NA	NA	NA	NA
43*^a^*	D610K*	0.1	H, M, U	5.5	NA	None	NA	NA	NA	NA	NA	NA	NA	NA
44	P392S	0.1	H, M	3.0	NA	None	NA	NA	NA	NA	NA	NA	NA	NA
45*^a^*	R841C	4.0	H	NK	NA	None	NA	NA	NA	NA	NA	NA	NA	NA

All variants are reported in the Androgen Receptor Genes Mutation Database^18^ except those marked with asterisks.

Abbreviations: *AR*, androgen receptor gene; B, bilateral undescended testes; DHT, dihydrotestosterone; EMS, external masculinization score; G, gynecomastia; Gy’mastia, gynecomastia; H, hypospadias; M, micropenis; N, no; NA, not applicable; NK, not known; Present^n^, presentation; Psy, clinical psychology support; Sx, surgery; T, testosterone; U, unilateral undescended testis; Y, yes.

^*a*^Cases with positive family history of androgen insensitivity syndrome.

^*b*^Case No. 12 and 13, gynecomastia resolved spontaneously at the ages of 21.4 and 15 years, respectively.

### Clinical Features at First Presentation

**Figure 2.**
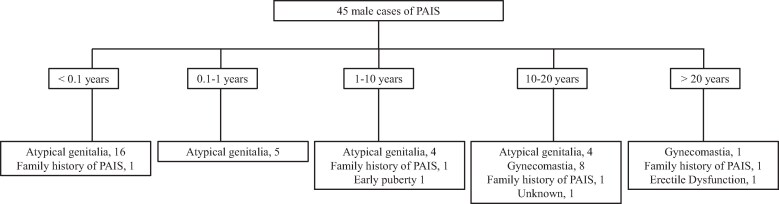
Description of presentation in the 46 genetically confirmed cases of partial androgen insensitivity syndrome according to age groups.

In the 45 cases with genetically confirmed PAIS, atypical genitalia was the presenting feature in 29 (64%), gynecomastia in 9 (20%), and family history of PAIS in 4 (9%) (Fig. 2). Details of atypical genitalia are presented in Table 1. The majority of the cases presented in infancy with the next commonest group in adolescence, who presented with gynecomastia (Fig. 2). The median age at presentation was 1.0 year (0.1, 26), and the EMS at first presentation was 6 (2, 12). Of the 9 PAIS cases where gynecomastia was the reason for presentation at the particular centers, 4 also had previous signs of DSD such as hypospadias, micropenis, and undescended testis.

### Onset of Gynecomastia

Of the 45 cases of PAIS, gynecomastia was reported over the follow-up period in 41 (91%). At last assessment, the median age of this group was 19.3 years (11.0, 65.3). The median age at onset of puberty and gynecomastia development were 12.0 years (10.5, 14.0) and 13.5 years (11.0, 29.0), respectively (*P* = .002). In the 4 (9%) cases that had not developed gynecomastia, the median age at last assessment was 15.7 years (10.6, 17.0) (*P* = .053). Median EMS at the time of gynecomastia development in the 23 cases where data were available was 9 (5, 12), and micropenis was also present in 77% of PAIS cases at the time of gynecomastia development. Among the 4 PAIS patients who had no gynecomastia, 2 (50%) had micropenis at last follow-up and the median EMS at last follow-up was 10.5 (3, 12). The 41 PAIS patients who developed gynecomastia had a total of of 42 *AR* variants and in 3 (7%) were located in NTD, 9 (21%) in DBD, and 30 (71%) in LBD. In the 4 patients with no gynecomastia, *AR* variants were located in NTD, DBD, and LBD in 1, 2, and 1 cases, respectively. There were no associations between the position of *AR* variants and the development of gynecomastia (*P* = .112).

#### Biochemistry at gynecomastia development and most recent assessment

**Figure 3.**
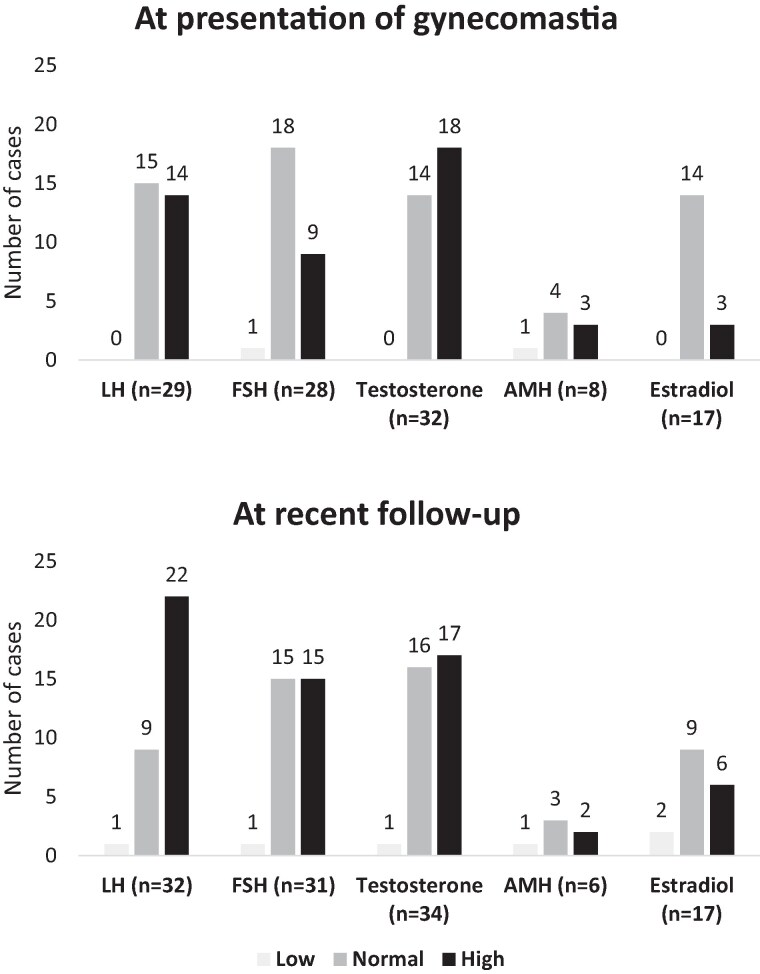
Biochemistry at presentation with gynecomastia and at most recent follow-up among genetically confirmed cases of partial androgen insensitivity syndrome. Normal, high, and low defined as hormone concentrations within normal, above the 95th percentile, and below the 5th percentile of reference rages for age and sex. Abbreviation: AMH, anti-Müllerian hormone.

At the development of gynecomastia, serum testosterone was reported to be raised in 18/32 (56%), and LH and FSH were raised in 14/29 (48%) and 9/28 (32%), respectively (Fig. 3). Of the 8 PAIS cases where serum anti-Müllerian hormone (AMH) concentration was available, 4 (50%) had normal serum AMH concentrations and serum estradiol concentration was within the normal male range in 14/17 (82%) cases (Fig. 3). Biochemical measurements at most recent assessment at a median age of 19 years (11.0, 55.4) revealed elevated serum testosterone in 17/34 (50%), elevated LH concentration in 22/32 (69%), elevated FSH concentration in 15/31 (48%), elevated estradiol concentration in 6/17 (35%), and elevated AMH in 3/6 (50%) cases. Only 3 out of 5 PAIS without gynecomastia had biochemical data available, and all 3 had high testosterone levels. One had normal LH and FSH, 1 had elevated LH and FSH, and the last 1 had low LH and FSH. The actual biochemical information in these cases is available in more detail in the supplementary table (15).

## Management of Gynecomastia

Details of the management of gynecomastia were available in 35/41 cases (85%) (Table 1). A wide range of drugs had been used to manage gynecomastia including transdermal testosterone (n = 3), 2.5% dihydrotestosterone gel (n = 2), tamoxifen (n = 3), letrozole (n = 3), and anastrozole (n = 1). Median age at the initiation of medical treatment was 13.9 years (12.0, 21.0). Other than 1 patient exhibiting weight gain after receiving letrozole, no adverse events were reported among those who received medical treatment. Twenty-five cases (71%) had either undergone surgery at a median age of 16.0 years (14.0, 23.0) or were awaiting surgery (n = 3), and 9 of these 25 (36%) had received prior medical therapy with poor response. The median clinician score for therapy effectiveness was reported as 10 (3, 10) in those who had undergone surgery compared to 3 (1, 8) for medical therapy (*P* < .0001). Of the 41, 7 (17%) did not receive any therapy; 3 cases were reported to have minimal gynecomastia, and in 2 cases (cases 12 and 13), who were siblings, therapy was not required because of spontaneous resolution at the age of 21.4 and 15 years, respectively. The reason for no therapy in the remaining 2 cases was unknown. Of the 31 cases where information was available, 13 (42%) had received clinical psychology support. The median age of receiving clinical psychology support was 12.9 years (11.0, 14.5), and the median clinician score for therapy effectiveness for clinical psychology was 5.0 (4, 8). No cases of gender incongruence were reported. Breast cancer screening as part of routine clinical care was reported to occur in 2 of 28 cases (7%) where the reporting center responded.

## Discussion

In adolescents and men with PAIS, gynecomastia has been reported in over 80% of cases where an *AR* variant has been confirmed (6, 8, 16, 17), and the current study of a larger cohort has confirmed this previous observation and highlighted the extent of variation in its natural history and management. On average, only 3 cases of PAIS older than 10 years at the time of the study were present at any participating center, and this confirms the rarity of male PAIS while emphasizing the need for pooling and exchanging data through a common platform, such as the I-DSD Registry.

Although a substantial proportion of the current cohort of PAIS cases had presented with gynecomastia, the prevalence of a pathological variant in *AR* remains unclear in adolescents and men who present with gynecomastia (18). The majority of *AR* variants in the current study have previously been described (19). However, the pathogenicity of some variants in this study may not have been fully established. Most variants were located in LBD, and there was no association between the location of the variant and the occurrence of gynecomastia. However, a more detailed analysis would require a larger sample size as well as further study of the pathogenicity of the variant. It has been suggested that those adolescents with gynecomastia who have elevated concentrations of testosterone, estradiol, and LH in puberty but normal FSH should be suspected of having PAIS and should undergo genetic testing of *AR* (6). Although this would be considered the classical biochemical picture of androgen insensitivity, there is increasing evidence that this does not discriminate sufficiently in the real-world setting (8). Indeed, in the current cohort, almost half of the cases did not have an elevated testosterone or LH concentration at the presentation of gynecomastia, the finding of a raised estradiol was very uncommon, and the FSH concentration was considered to be raised above the normal range for the center in almost half of the cases. Previous studies in androgen insensitivity syndrome have reported that a small proportion of boys may display a poor testosterone response to hCG stimulation (20), suggesting that in some cases of androgen insensitivity syndrome there is coexisting primary testicular insufficiency (17, 21, 22), possibly due to cryptorchidism or as a postsurgical sequelae of multiple orchidopexies. The current study also suggests that gynecomastia can occur in the absence of increased circulating estradiol and that tissue androgen resistance may play an important role.

Unlike other forms of gynecomastia, where exogenous administration or endogenous peripubertal synthesis of testosterone may alleviate the condition, the gynecomastia that is associated with PAIS does not seem to resolve and may require intervention. However, there are concerns about performing irreversible interventions in a group of young people who may want to consider gender reassignment in the future. Psychological distress in PAIS, arising from both sexual dissatisfaction and disappointment with externally undervirilized appearance (17, 23, 24), has previously been reported, and it is possible that the gynecomastia may itself contribute to this distress. In the current study, over a third of cases received clinical psychology support as part of their gynecomastia management, and there were no reported cases of gender incongruence.

Three possible categories of drugs have been considered for the treatment of pathologic gynecomastia: (1) selective estrogen receptor modulators such as tamoxifen, raloxifene, and clomiphene citrate; (2) aromatase inhibitors such as testolactone, anastrozole, and letrozole; and (3) androgens. However, current data are insufficient to demonstrate the effectiveness of any of these drugs for the treatment of pubertal gynecomastia (1). The data on medication use for treating gynecomastia in PAIS patients is even more limited. A single report of tamoxifen in 2 brothers with PAIS suggested that tamoxifen was effective (25). Tamoxifen has often been used for treating pubertal gynecomastia, and favorable outcomes have been reported (26, 27). There were 3 cases of PAIS in the current study who were treated with tamoxifen with little effectiveness, and all 3 proceeded to surgery. Four cases received aromatase inhibitors (3 letrozole and 1 anastrozole), with variable response, and 2 of these cases proceeded to surgery. Aromatase inhibitors have also been used for treating pubertal gynecomastia, and it appears that their effectiveness may be lower than that of selective estrogen receptor modulators or may not differ from placebo (28, 29). Five cases of PAIS in the current study were treated with transdermal androgens, either transdermal testosterone or dihydrotestosterone. However, 4 out of these 5 eventually proceeded to surgery. The use of nonaromatizable androgens such as dihydrotestosterone would be preferable to testosterone, which may theoretically worsen gynecomastia in PAIS and would need to be used cautiously, perhaps in combination with aromatase antagonists. In summary, of the 12 cases who had medical treatment, 9 eventually had surgery. There are several case reports of surgical mastectomy among adolescents and men with PAIS (6, 8, 10, 17, 21, 30), and, in general, in the current cohort the results confirm the effectiveness of this therapeutic approach and suggest that correcting the imbalance of estrogen to androgen ratio is not as effective as surgery in this condition. This would suggest that the primary mechanism of gynecomastia in PAIS does not arise from hormonal imbalance but rather from a lack of androgen action in inhibiting breast development. Our findings also concur with the general consensus on the relative merits of medical and surgical interventions for gynecomastia in conditions other than PAIS (2).

Although it is not completely clear whether gynecomastia itself or the underlying factors contributing to gynecomastia are causative factors for breast cancer in men (31), it is possible that it is the men who have an elevated estrogen status who have an increased risk (32, 33). Androgens also exert an antimitogenic effect on breast tissue by inhibiting tissue growth in the breast (34), so it is also possible that young men with PAIS and gynecomastia may be predisposed to male breast cancer (35, 36). Breast cancer has been reported in men with PAIS, and its risk may increase with increasing age (35, 37-39). Given that the median age of the current cohort was relatively young, there is a need to follow PAIS men with gynecomastia over a longer period. The *AR* variants in the 3 cases that were reported to have breast cancer were located in the DNA binding domain (R608Q, R609K), which is known to impact the transcriptional activity of *AR* (40). Currently, there are no screening programs for men who are considered to be at high risk of breast cancer (41), and less than 10% of PAIS cases in the current study had undergone breast cancer screening. However, it would be prudent to advise young men with PAIS and gynecomastia to include breast tissue palpation as part of regular annual physical examinations.

This is the largest cohort of PAIS characterizing the development of gynecomastia and its management in males with genetically proven PAIS using data from the I-DSD Registry database. Previous PAIS cohorts (6, 8, 16, 17) have described the occurrence of gynecomastia in PAIS but have not have explored the details as comprehensively in terms of clinical and biochemical aspects and the relationship with the *AR* variant as in the current study. Moreover, this study also gathers information on the management and efficacy of various treatment modalities for gynecomastia in PAIS, a topic that has not been studied in-depth before and lacks international consensus. We were unable to collect data from all centers in the I-DSD Registry, and it is therefore possible that this may have introduced some selection bias; this will need to be investigated in future prospective studies.

Lastly, the study shows the utility of the I-DSD Registry for studying an important clinical outcome in a real-world setting in people with a very rare condition such as PAIS. The study also highlights the need for standardizing the clinical assessment prior to, as well as following, the interventions and the need for long-term follow-up into adulthood. The study used an improvised assessment of clinical effectiveness, but it would have been more informative to understand the views of the patients themselves before and after the intervention through a validated questionnaire (42).

In summary, gynecomastia is a common clinical finding in boys and men over 10 years old with PAIS. A surgical approach to therapy seems to be more effective than medical therapy. However, there is a need for a more systematic approach and further studies into adulthood to understand the long-term effectiveness of the current approach.

## Data Availability

All the relevant data underlying this article are available in the article and in its online supplementary material (15). Additional data will be shared on reasonable request to the corresponding author.
